# Intravitreal anti-VEGF treatment for choroidal neovascularization secondary to traumatic choroidal rupture

**DOI:** 10.1186/s12886-019-1242-7

**Published:** 2019-11-26

**Authors:** T. Barth, F. Zeman, H. Helbig, M.-A. Gamulescu

**Affiliations:** 1 Department of Ophthalmology, University Medical Centre Regensburg, Franz-Josef-Strauß-Allee, 11 93053 Regensburg, Germany; 2 Centre for clinical studies (ZKS), University Medical Centre Regensburg, Franz-Josef-Strauß-Allee, 11 93053 Regensburg, Germany

**Keywords:** Secondary choroidal neovascularization, Choroidal rupture, Intravitreal anti-VEGF (vascular endothelial growth factor) treatment

## Abstract

**Background:**

So far only single cases with short follow-up have been reported on the use of intravitreal anti-VEGF for traumatic choroidal neovascularizations (CNV). This paper reports a large case series of patients with CNV secondary to choroidal rupture after ocular trauma receiving intravitreal anti-VEGF (vascular endothelial growth factor) injections.

**Methods:**

Fifty-four patients with unilateral choroidal rupture after ocular trauma diagnosed between 2000 and 2016 were retrospectively evaluated. Eleven patients with CNV secondary to choroidal rupture were identified. Five eyes with traumatic secondary CNV were treated with anti-VEGF and were systematically analysed. The other 4 patients with inactive CNV underwent watchful observation.

**Results:**

Four men and one woman with a mean age of 29 years (SD 12.4; range 19–45) had intravitreal anti-VEGF therapy for traumatic CNV. Another 4 patients with a mean age of 37 years (SD 6.6; range 31–46) presented with inactive CNV and did not receive specific treatment. In all 9 cases the mean interval between the ocular trauma and the diagnosis of CNV was 5.7 months (SD 4.75; range 2–12). In the treatment group per eye 4.2 injections (SD 3.2; range 1–8) were given on average. Four eyes were treated with bevacizumab and one eye with ranibizumab. Regression of CNV was noted in all eyes. In 4 eyes visual acuity (VA) improved, one eye kept stable visual acuity.

**Conclusions:**

Here, we present the up to now largest case series of traumatic CNV membranes treated with anti-VEGF injections with a mean follow-up period of 5 years. Intravitreal anti-VEGF therapy seems to be safe and effective for secondary CNV after choroidal rupture. Compared to exudative age-related macular degeneration fewer injections are needed to control the disease.

**Trial registration:**

Retrospective registration with local ethics committee on 21 March 2019. Trial registration number is 19-1368-104.

## Introduction

Traumatic choroidal neovascularization (CNV) occurs in patients with choroidal rupture following severe ocular injuries [1]. Compared to CNV in age-related macular degeneration (AMD) the course of traumatic neovascular membranes is relatively benign [1]. As choroidal neovascularizations are a consistent part of the ocular repair mechanism they usually regress spontaneously as part of the healing process [2, 3]. The formation of persistent CNV with substantial loss of central vision happens in 5-25% of eyes with choroidal ruptures [1, 3]. In the early phase (within 6 months of the injury) the CNV development is explained by an exuberant repair reaction or just persistence of the normal reparative neovascular tissue [1]. Late CNV formation (after one year of the injury) could either be caused by awakening of an old dormant CNV or more likely by a new CNV in an area of local weakness [1]. In earlier days selected case of traumatic CNV were treated by photodynamic therapy (PDT) [4, 5], laser photocoagulation [6] or submacular surgery [7]. After the introduction of intravitreal anti-VEGF (vascular endothelial growth factor) as therapy for exudative AMD some reports of antiangiogenic injections for CNV of other causes than AMD [8–10] and in younger populations [11] have been published. So far no larger only single case reports have been published on intravitreal injections for traumatic CNV [12–14]. Thus, it is important to collect data about the use of intravitreal anti-VEGF agents in individuals with this condition.

## Methods

We identified 54 cases of choroidal rupture after ocular trauma, which were diagnosed between 2000 and 2016 at a large tertiary university eye hospital. Eleven patients with CNV secondary to choroidal rupture were identified and further evaluated. We divided the study population in two different groups. The first group (*n* = 5) included patients with active CNV lesion at initial presentation, that received intravitreal anti-VEGF injections. The second group (*n* = 4) consisted of eyes with inactive CNV at first presentation which did not receive specific treatment. The two remaining patients were excluded because of different treatment strategies (vitrectomy, PDT). The patients' medical records were retrospectively analysed. The following parameters were assessed: age, sex, type of injury, interval between trauma and CNV formation, treatment modalities and rate of recurrence. For anonymization the patients were grouped into different age groups (18-25 years, 26 -30 years, 31 - 40 years, 41 - 50 years.) The baseline examination included measurement of best corrected visual acuity (BCVA), slit lamp examination, ophthalmoscopy and retinal imaging. Diagnosis of CNV secondary to choroidal rupture was made clinically by at least two retina specialists and was confirmed by multimodal imaging of the posterior segment. ) Fundus fluorescein angiography (FFA) was achieved with Spectralis® (Heidelberg Engineering, Heidelberg, Germany). On the basis of the FFA images the greatest linear dimension (GLD) of the lesion was calculated and the type, activity and location of the CNV was analysed (subfoveal: within the foveal avascular zone (FAZ), juxtafoveal: distance from FAZ ≤ 200 μm, extrafoveal: distance from FAZ > 200 μm). The decision of the treatment modality was made by at least two independent ophthalmologists and was based on the activity of the CNV lesion verified by leakage on FFA images. For the patients that underwent no specific CNV treatment no further optical coherence tomography (OCT) scans were performed. For the patients that underwent anti-VEGF treatment retinal imaging included also optical coherence tomography (OCT) at baseline and on all follow-up visits with a central 6-line radial OCT. The central foveal thickness (CFT) was calculated and the existence of intra- or subretinal fluid was noted. The definition of recurrence was reappearing activity either seen by leakage on FFA images or fluid on OCT scans at least three months after the last injection. The patients in the treatment group received at least one anti-VEGF injection of either bevacizumab (1.25 mg/0.05 ml) or ranibizumab (0.5 mg/0.05 ml). There was no standardised treatment protocol. In every case the count, frequency and substance of anti-VEGF injections was chosen individually. The follow-up examination included Snellen's charts visual acuity, slit-lamp examination, funduscopy and retinal imaging. A conversion of the visual acuity equivalents attained using Snellen's charts to logMAR values was performed for statistical evaluation. The statistical investigation was achieved by using SPSS 23.0 (IBM, Chicago, USA). Categorical variables are listed as frequency counts and percentages. Continuous values are displayed as mean ± standard deviation and range (min-max); Mean values were compared using Student’s t-test. A p-value <0.05 was considered statistically significant.

## Results

### Demographic and clinical data

Overall, 54 patients were diagnosed with choroidal rupture after ocular trauma between 2000 and 2016. Eleven (20%) of these patients developed secondary CNV. One patient in 2001 was treated with focal laser photocoagulation and PDT before the introduction of intravitreal anti-VEGF. Another patient underwent pars-plana-vitrectomy for CNV-related vitreous haemorrhage. Of the 9 remaining cases, 5 eyes diagnosed between 2009 and 2016 with secondary traumatic CNV received intravitreal antiangiogenic treatment. In the 4 other eyes no specific medical treatment was recommended because of inactivity of the lesion at initial presentation. Overall 6 (67%) right eyes and 3 (33%) left eye were assessed. The study population involved 6 (67%) men and 3 women (33%). The mean age was 32 years (SD 10.3; range 19-46). Eight patients had a history of blunt trauma with one case of multiple facial fractures. The ninth patient presented with choroidal rupture after a penetrating eye injury with an intraocular foreign body. In every case the CNV development occurred within the early phase of one year after injury. The mean interval between the ocular trauma and the diagnosis of CNV was 5.7 months (SD 4.75; range 2-12). At baseline three patients reported decreased central vision with an average duration of symptoms of 10 weeks (SD 12; range 2-24). Six patients were diagnosed with CNV during posttraumatic routine follow-up examination. Table 1 summarizes the demographic and clinical data of the treatment group and the group without therapy.

**Table 1:** Clinical data of patients with traumatic choroidal neovascularization (CNV) treated with anti-VEGF [Group 1] and patient without CNV treatment [Group 2]

Patients with traumatic CNV	Group 1 (intravitreal anti-VEGF)	Group 2 (no treatment)	Both groups
No. of cases	5	4	9
Right eyes	4 (80%)	2 (50%)	6 (67%)
Age (years)	29 ± 12.4	37 ± 6.6	32 ± 10.3
Sex (men)	4 (80%)	2 (50%)	6 (67%)
Time between trauma and CNV (months)	6.0 ± 4.6	5.3 ± 5.9	5.7 ± 4.75
GLD of the CNV (μm)	1345 ± 865	1215 ± 535	1296 ± 717
Initial logMAR BCVA	0.80 ± 0.32	1.2 ± 0.35	0.98 ± 0.38
Final logMAR BCVA	0.42 ± 0.26	1.1 ± 0.35	0.66 ± 0.43
Follow-up (months)	62 ± 32	82 ± 66	69 ± 44

*BCVA* Best corrected visual acuity, *GLD* Greatest linear diameter, *CFT* Central foveal thickness

Data present mean ± SD or absolute number (percentage)

### Morphologic and anatomical results

**Fig. 1:**
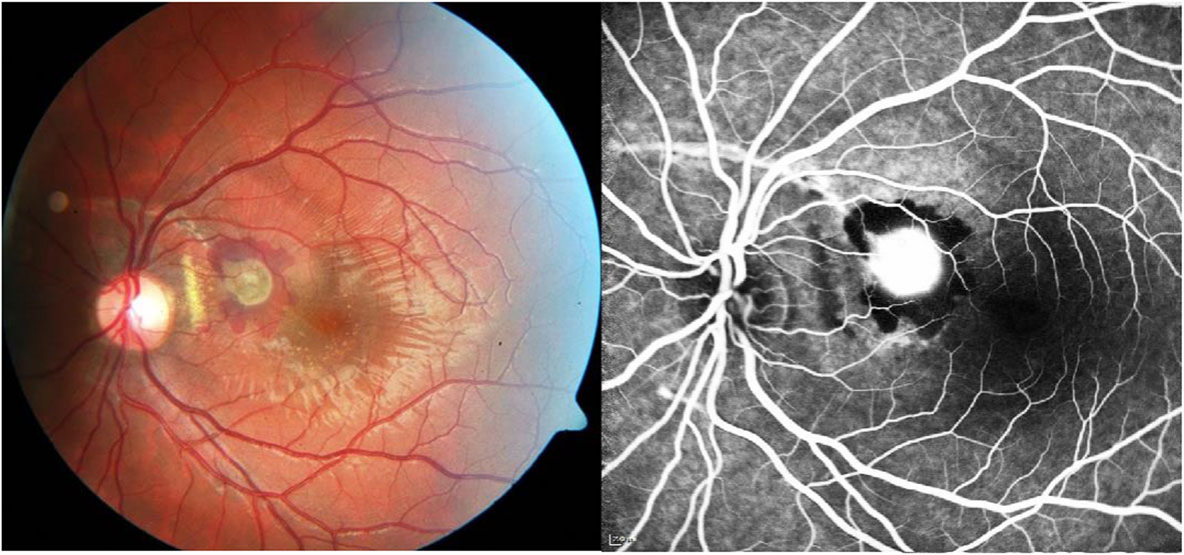
Traumatic CNV in a 19-year-old male. Colour image and fundus fluorescein angiography (FFA) of a 19-year-old man with traumatic choroidal neovascularization (CNV) in the left eye. Arcuate choroidal rupture can be seen under-crossing the vessels above the optic disc. The greyish CNV with ambient subretinal bleeding, located in the papillomacular region, shows significant leakage on FFA.

**Fig. 2:**
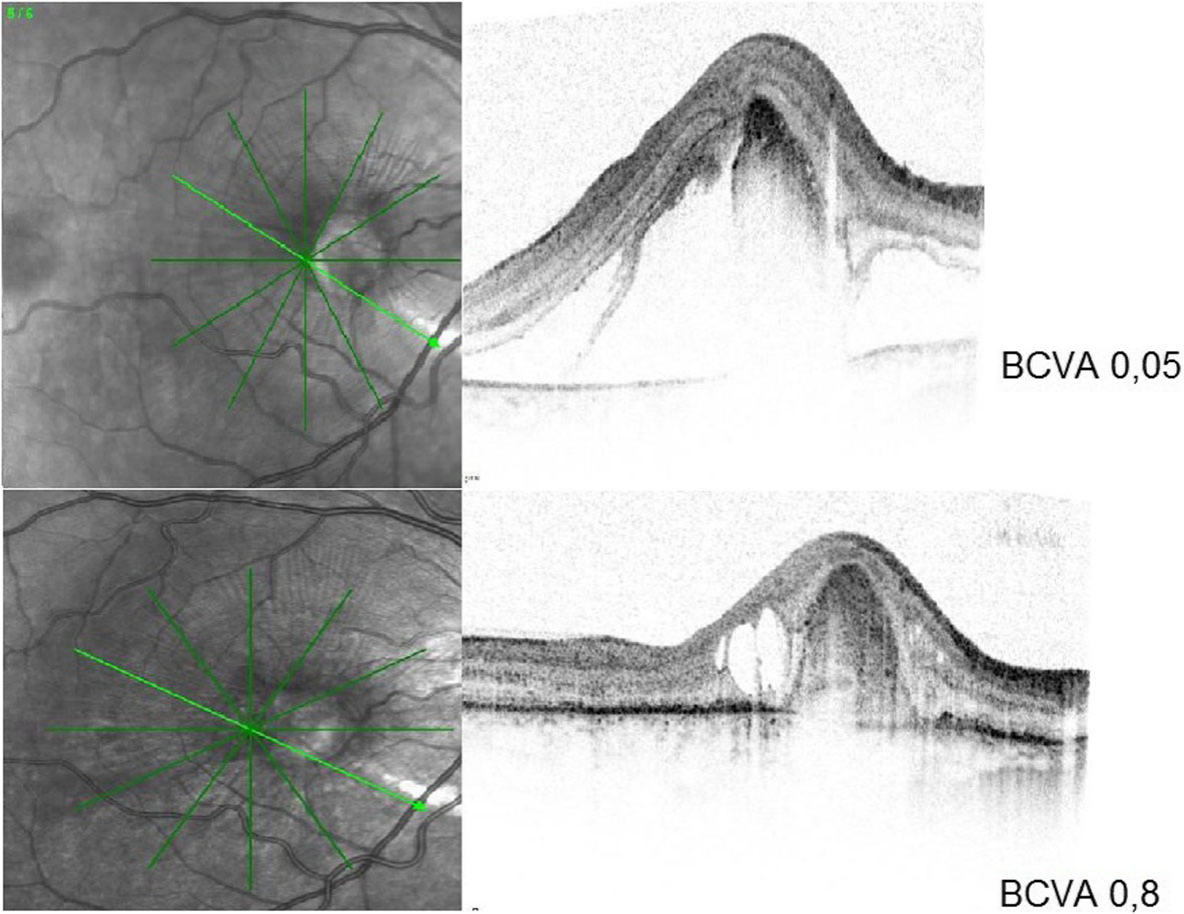
Regression of traumatic CNV in a 23-year-old male treated with anti-VEGF. Optical coherence tomography images of the right eye of a 19-year-old man with traumatic choroidal neovascularization (CNV) a) before treatment and b) four weeks after the last Bevacizumab injection showing regression of CNV. BCVA = best corrected visual acuity on day of retinal imaging

At the initial diagnosis all 9 eyes presented with a classic CNV lesion. In 4 eyes the CNV was located extrafoveally (44%), three CNV membranes were in the subfoveal region (33%) and 2 patients showed juxtafoveal location (22%). Four eyes (44%) with an inactive and scarred CNV lesion at initial presentation underwent watchful observation and did not receive specific medical treatment. Five eyes (56%) showed manifest CNV activity detected by apparent leakage on FFA images and were treated with intravitreal anti-VEGF injections. In these eyes with CNV the baseline OCT scan showed a mean CFT of 645 μm (SD 359, range 329-1240). After completion of anti-VEGF-therapy an average CFT of 403 μm (SD 145, range 214-586) was measured at the last visit. This reduction of CFT after anti-VEGF treatment was not statistically significant (p= 0.089). FFA was performed in all 9 eyes and showed a mean GLD of the neovascular membrane of 1296 μm (SD 717 μm, range 451-2725). Details about the CNV morphology in the treatment group as well are listed in Table 2. Figure 1 shows fundus and FFA images of a 19-year-old male with traumatic CNV in the left eye. The OCT scans of a 23-year-old man with traumatic CNV in the right eye before treatment and 4 weeks after the last injection are presented in Fig. 2.

**Table 2:** Morphologic characteristics of 5 traumatic choroidal neovascularizations (CNV) treated with intravitreal anti-VEGF-injections

No.	Location of choroidal rupture	CNV type	CNV location	CNV activity	GLD (μm)	Initial CFT (μm)	Final CFT (μm)
1	~ 1 PD above optic disc	classic	extrafoveal	active	451	568	434
2	~ 1 PD under optic disc	classic	extrafoveal	active	1124	1240	586
3	~ 1 PD above optic disc	classic	extrafoveal	active	890	329	307
4	~ 1 PD temporal optic disc	classic	subfoveal	active	1535	410	214
5	~ 1 PD temporal optic disc	classic	subfoveal	active	2725	678	475

*GLD* Greatest linear diameter, *FFA* Fundus fluorescein angiography, *CFT* Central foveal thickness, *VEGF* Vascular endothelial growth factor

### Treatment characteristics and recurrence rates

The 5 patients with an active CNV lesion received intravitreal antiangiogenic treatment as primary treatment for CNV. On average, 4.2 injections (SD 3.1, range 1-8) were given per eye. Four eyes were treated with bevacizumab, 1 eye with ranibizumab. The decision about the injection scheme was made individually and was dependent on the size and activity of the lesion. In two (40%) eyes, which received a single anti-VEGF load at the beginning, this single injection was sufficient to control the disease. The other 3 (60%) eyes were treated with an upload of three monthly injections. After a three month treatment pause 2 of these patients needed another 2 or 3 injections to control the CNV activity over a period of 3 to 6 months. In one of these patients recurrence occurred after a six-month treatment pause and was controlled by a single re-injection. The third patients with an initial upload of three injections had a two month treatment pause and another five injections afterwards over a period of half a year.

### Functional outcome in the treatment group

Before the anti-VEGF treatment the mean logMAR BCVA in the treatment group was 0.80 (SD 0.32, range 0.4-1.3), which equates to a decimal visual acuity (VA) of 0.16. The logMAR BCVA in the cases without further therapy was 1.2 (SD 0.35, range 0.7-1.5) equivalent to a decimal VA of 0.06. As expected the group of patients with an inactive, scared CNV had a worse VA at baseline than the eyes of the treatment group. After the anti-VEGF therapy an average logMAR BCVA of 0.42 (SD 0.26, range 0.1-0.8) was assessed in the treatment group, which equates to a decimal VA of 0.4. Every CNV responded well to anti-VEGF treatment and all eyes presented with improved (*n* = 4, 80%) or stable VA (*n* = 1, 20%) after the last anti-VEGF injection. The mean change of BCVA was + 3.8 lines (SD 4.8; range 0-12). Nevertheless, the difference between baseline VA and final VA was statistically not significant (*p* = 0.152). In the group without treatment the mean logMAR BCVA was 1.1 (SD 0.35, range 0.7 -1.4) at the last follow-up appointment. There was no significant change between baseline and follow-up VA in this group (*p* = 0.939). The follow-up period was 62 (SD 32; range 17 - 102) months in the treatment group and 82 months (SD 66; range 6 - 120) in the group without therapy. Table 3 gives a detailed overview of the clinical parameters of the treatment group.

**Table 3:** Clinical parameters of 5 eyes with traumatic choroidal neovascularization (CNV) treated with intravitreal anti-VEGF-injections

No.	age group (yr)	eye	Type of trauma	Anti-VEGF drug	No. of injections	Initial logMAR BCVA	Final logMAR BCVA	ΔBCVA (lines)
1	18–25	RE	Penetration	Ranibizumab	6	0.20	0.3	+ 2
2	18–25	RE	Contusion	Bevacizumab	8	0.05	0.8	+ 12
3	18–25	LE	Contusion	Bevacizumab	1	0.16	0.40	+ 4
4	31–40	RE	Contusion	Bevacizumab	1	0.40	0.50	+ 1
5	41–50	RE	Contusion	Bevacizumab	5	0.16	0.16	± 0

*yr* Years, *RE* Right eye, *LE* Left eye, *CNV* Choroidal neovascularization, *BCVA* Best corrected visual acuity, *F/U* Follow-up, *VEGF* Vascular endothelial growth factor

## Discussion

Traumatic choroidal ruptures are seen in 5-10 % of patients with blunt ocular injuries [15, 16]. The growth of a secondary trauma-related CNV is a common complication in such eyes [1]. In our study population 20 % of all patients with traumatic choroidal rupture developed a CNV, which is in agreement with other publications [3, 15]. In line with other authors [15], we also had a young study population developing traumatic CNV with an average age of 32 years. Consistent with our data most patients with traumatic chorioretinopathies are male (up to 90%) [15].

Not every traumatic CNV needs to be treated [15]. The development of fibrovascular tissue and neovascularisation is part of the expected healing process and some CNV membranes can undergo watchful observation while regressing [2, 3, 15]. This is consistent with our report of 4 of 11 CNV membranes (36 %) that did not receive further ophthalmic treatment because of inactivity and scarring of the lesion at initial presentation. However, in cases of symptomatic CNV development and activity of the lesion verified by FFA imaging prompt treatment is indicated. Before the anti-VEGF aera secondary CNV membranes were treated with laser photocoagulation [6], photodynamic therapy [4, 5] or vitreoretinal surgery [7]. There is nowadays consensus that secondary CNV membranes should receive intravitreal anti-VEGF treatment as first-line-therapy [17].

To our knowledge, only single case reports have been published so far on intravitreal anti-VEGF therapy for traumatic CNV in the past [12–14]. In these cases one injection of either ranibizumab [12] or bevacizumab [13, 14] was administered. However, the follow-up period with a range of 6 to 12 months was quite short compared to our study. This fact could explain the low number of injections given in these individual cases. In our series one case of late recurrence after a six months treatment pause occurred, which emphasizes the need of a thoroughly and adequate long follow-up on these patients. There are also larger retrospective studies with up to 21 cases of intravitreal antiangiogenic treatment of neovascular membranes in younger patients (< 50 years of age) and from other causes than AMD [8–10]. So far, the MINERVA study is the largest prospective phase III trial to evaluate ranibizumab for treatment of CNV of uncommon cause. In this study the group of choroidal neovascularization of miscellaneous etiology included 6 patients with choroidal rupture and 1 with posttraumatic etiology, but no separate evaluation of the traumatic CNV group is available [18].

In our patient population with secondary traumatic CNV intravitreal anti-VEGF therapy was effective in the treatment group in respect of functional and anatomical outcome. In every case a good response to anti-VEGF therapy was noted by CNV regression documented on OCT and FFA imaging. In all treated eyes the CFT was reduced compared to the baseline examination. Every eye showed an improved (*n* = 4) or stable (*n* = 1) BCVA with an average gain of 3.8 BCVA lines. Although the improvement in BCVA (*p* = 0.152) and reduction of CFT (*p* = 0.089) was not statistically significant due to the low sample size, we consider the difference of mean values of BVCA and CFT between baseline and final visit as clinically relevant. As expected in the group without anti-VEGF treatment no significant change was noted between baseline and follow-up VA (*p* = 0.939).

Overall, we conclude that in younger individuals with secondary traumatic CNV the response to intravitreal antiangiogenic treatment seems to be more favourable than in older patients with wet AMD [19]. Congruent with our data, the average count of anti-VEGF injections required to control the activity of the disease is lower compared to patients with AMD-related CNV [18]. Although good results have been achieved with intravitreal antiangiogenic drugs, the lack of long-term data regarding safety and systemic side effects in young individuals has to be emphasized. However, in our study population no serious ocular or systemic side effects were recorded.

## Conclusions

We present the up to now largest retrospective case series with a systematic review of patients with secondary traumatic CNV receiving intravitreal anti-VEGF agents with a mean follow-up of five years. The limitations of our study are its retrospective design, the low sample size, the lack of a standard protocol (different injection regimes and anti-VEGF agents) and the monocentric character. The sample size is compared to other single case reports quite good and every case was well documented including retinal imaging. Further clinical trials with a prospective and randomized character are unlikely to be published with refer to the rarity of this condition.

## Data Availability

Supporting data can be accessed by contact to the corresponding author (teresa.barth@ukr.de). The datasets analysed are available from the corresponding author on reasonable request.

## Abbreviations

AMD: Age-related macular degeneration
BCVA: Best corrected visual acuity
CFT: Central foveal thickness
CNV: Choroidal neovascularization
FAZ: Foveal avascular zone
FFA: Fundus fluorescein angiography
GLD: Greatest linear dimension
OCT: Optical coherence tomography
PDT: Photodynamic therapy
VEGF: Vascular endothelial growth factor
